# Inducible Clindamycin-Resistant *Staphylococcus aureus* Strains in Africa: A Systematic Review

**DOI:** 10.1155/2022/1835603

**Published:** 2022-04-19

**Authors:** Muluneh Assefa

**Affiliations:** Department of Medical Microbiology, School of Biomedical and Laboratory Sciences, College of Medicine and Health Sciences, University of Gondar, P.O. Box 196, Gondar, Ethiopia

## Abstract

**Introduction:**

Excessive use of clindamycin enhances the acquisition of inducible clindamycin-resistant *S. aureus* strains, which is a significant health problem in Africa. The main objective of this review study was to determine the prevalence of inducible clindamycin resistance and related genes among *S. aureus* isolates in Africa.

**Methods:**

A qualitative systematic review was conducted on inducible clindamycin resistance among *S. aureus* isolates in Africa using electronic databases such as Google Scholar and PubMed. Articles published in English before 2021 were selected, and relevant data were extracted, collected, and analyzed.

**Results:**

In our search, 22 articles met the eligibility criteria for this review study. Of 3064 total *S. aureus* isolates, 605 had iMLSB phenotype. The overall prevalence of inducible clindamycin resistance in *S. aureus* isolates was 19.8% with a range of 2.9% to 44.0%. A high number of iMLSB phenotypes were observed in MRSA isolates (3.6–77.8%) than MSSA (0–58.8%). The overall prevalence of the iMLSB phenotype in MRSA strains was 26.8% (279/1041). The maximum peak prevalence of inducible clindamycin resistance among *S. aureus* isolates recorded in the continent was 44.0% in Egypt, followed by 35.8% in Libya and 33.3% in Uganda in 2017, 2007, and 2013, respectively. The highest prevalence of iMLSB phenotype in MRSA strains was reported in Egypt, 77.8%, followed by Nigeria, 75.0%, and Libya, 66.2%. Among the recovered drug-resistance genes, ermA, ermC, and msrA genes were commonly detected in Egypt with 67.9%, 70.0%, and 70.0% prevalence, respectively.

**Conclusion:**

This review highlights a higher inducible resistance of *S. aureus*, including MRSA strains to clindamycin in the continent. Regular screening of these strains, wise use of clindamycin, and molecular detection and genotyping of resistant genes are urgent.

## 1. Introduction

*Staphylococcus aureus* is normally found in human skin and mucous membranes. It is a common human pathogen that causes skin and soft-tissue infections, abscesses, pneumonia, osteomyelitis, endocarditis, arthritis, and sepsis in both the community and hospital environment, and the spread of methicillin-resistant *Staphylococcus aureus* (MRSA) through the acquisition of highly transmissible mecA/mecC genes has made treatment difficult [1, 2].

Even though the global average incidence of MRSA is 40.0%, reports from African countries reveal rates ranging from 12.0 to 80.0%, with some countries exceeding 82.0% [3, 4]. Antimicrobial resistance has become a severe health hazard worldwide, and its burden has increased in Africa because of a highly infectious disease burden, poor hygiene, lack of environmental sanitation, and poor infection control. The treatment of MRSA infections in African nations is problematic due to the lack of antibiotics with proven efficacy [5]. The rising prevalence of community-acquired MRSA has sparked interest in using macrolide-lincosamide-streptogramin (MLSB) antibiotics, particularly clindamycin to treat *S. aureus*-associated pneumonia and skin and soft-tissue infections [6].

Clindamycin is the chosen antibiotic because of its superior pharmacokinetics, availability in intravenous and oral formulations with 90% oral bioavailability, low cost, strong tissue penetration, accumulation in deep abscesses, and capacity to inhibit toxin generation in *S. aureus* [7]. Excessive use of clindamycin, on the other hand, enhanced the acquisition of inducible resistance, leading to therapeutic failure [8]. The main mechanisms of resistance in the MLSB drugs include target site alteration, efflux pump expression, and mutation [9]. The MLSB phenotype can be either constitutive (cMLSB phenotype) in which rRNA methylase is always produced) or inducible (iMLSB phenotype) in which methylase is produced only when an inducing substance like erythromycin is present. During treatment, iMLSB phenotypes can be mutated into cMLSB phenotypes [10]. Owing to this, the Clinical and Laboratory Standards Institute (CLSI) recommends using the double-disk diffusion method (*D*-test) for detecting inducible resistance to clindamycin among *Staphylococcus aureus* isolates [11].

In Africa, *S. aureus* is becoming increasingly resistant to clindamycin due to clinicians' rash use of antibiotics without performing *D*-test and lack of laboratory facilities for molecular approaches such as polymerase chain reaction (PCR) based resistance gene detection. Although there has been published research on inducible clindamycin resistance, little is known about its dissemination and clinical significance in Africa, necessitating a compilation of data from the continent. To fill this gap, this systematic review provides an updated summary and valuable data. Therefore, this study mainly aimed to determine the prevalence of inducible clindamycin resistance and related resistance genes among *S. aureus* isolates in Africa.

## 2. Methods

### 2.1. Literature Search Strategy in Databases

**Figure 1:**
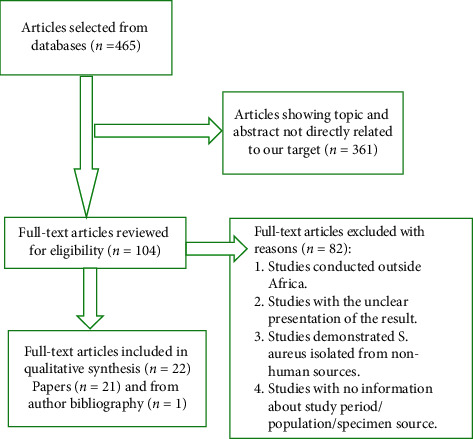
Flow diagram showing the procedure of eligible study selection to undergo review.

A systematic literature search was performed on published articles for inducible clindamycin resistance among *S. aureus* isolates in Africa with a study period before 2021 using electronic databases such as PubMed and Google Scholar. The following keywords were used with the help of Boolean operators: “inducible clindamycin resistance” OR “macrolide-lincosamide-streptogramin B resistance” OR “*D*-test” AND (“*Staphylococcus aureus*” OR “*S. aureus*” OR “methicillin-resistant *Staphylococcus aureus*” OR “methicillin-sensitive *Staphylococcus aureus*” OR MRSA OR MSSA) AND (Africa). The references of included articles were appropriately scanned to access related articles of interest. The literature search was not limited to a specific publication or year of study. In this review, we considered all studies that described inducible clindamycin resistance in *S. aureus* obtained from any type of human study participant in Africa. The procedure of eligible study selection is demonstrated in Figure 1.

### 2.2. Study Selection and Eligibility Criteria

All studies from Africa reported the following information in the full-text selected:

- Articles performed D-test for detecting the iMLSB phenotype in *S. aureus* according to CLSI guideline
- All articles published with a study period until 2021
- Articles published and written in English
- Articles with well-defined objectives and methodology
- Articles from a human source of specimen
- Articles including data on the number of *S. aureus* isolates and any source of specimen used
- Studies which investigated antibiotic resistance genes using PCR were also summarized

Articles that lacked all or most of the above variables, such as abstract only, not in English, duplicate reports, ambiguous results, and articles with overlapping data, were excluded.

### 2.3. Assessment of the Study Validity

The validity of each study was illustrated by the use of the selection and eligibility criteria described above, thereby excluding studies that have unclear results, are unrepresentative of the human population, or studies with noncomparable data. Studies vary in specimen source, and the human study population was not excluded.

### 2.4. Data Extraction and Collection

Essential data were extracted from eligible studies using Excel spreadsheet format, and any discrepancies were handled by the author. The following information was extracted from the selected studies: the percentage of iMLSB phenotype detected, the number of *S. aureus* isolates identified, the study period, the study population, geographic area where the study was conducted, the source of the specimen, the method of detection, the coexistence of antibiotic resistance genes, and the references were all considered.

### 2.5. Data Synthesis and Analysis

The data were synthesized qualitatively. Because of the relatively small number of studies used, inconsistencies between studies, and heterogeneity of the study populations between countries, we did not perform a quantitative synthesis. Data were summarized in the extraction table and analyzed manually. The overall prevalence of inducible clindamycin resistance in *S. aureus* or MRSA strains was calculated using the following formula:

(1) $$\begin{array}{c} \text{overall prevalence}=\frac{\text{sum of the iMLSB phenotypes}}{\text{total number of }S.\;aureus/\text{MRSA}}*100\%. \end{array}$$

According to the United Nations list of 54 African countries, the map of Africa was created using the website (https://mapchart.net/). Finally, charts were created using the Excel 2019 software.

## 3. Results

### 3.1. Literature Search

In electronic database searches, 465 articles were retrieved. After removing duplicates, 361 articles were avoided based on their titles and abstracts. The full-text articles of the remaining 104 articles were reviewed in detail for eligibility. Of these, 82 articles were discarded after the full-text had been reviewed for appropriate methodology, study population, the source of the specimens, clear result, and standard microbiological technique. Finally, 22 articles were included in the synthesis of the review (Figure 1).

### 3.2. General Characteristics of the Studies Included in This Systematic Review

The main characteristics of the 22 studies from 8 African countries included in this systematic review have been summarized in Table 1. All the studies used *D*-test for detecting inducible clindamycin resistance in *S. aureus* isolates, but some of them also used PCR for confirming the MLSB resistance genes. Studies used various specimens from human sources including swabs such as nasal, vaginal, cervical, urethral, wound, throat, ear, eye, and palm, respiratory specimens such as sputum, bronchoalveolar lavages, and tracheal aspirates, pus, urine, catheter, blood, semen, cerebrospinal fluid, pleural fluid, ascetic fluid, synovial fluid, and others. In this systematic review, most of the studies were conducted in clinical patients admitted to hospitals or outpatients, and the rest were conducted in healthy individuals with *S. aureus* carriers. Out of six studies that performed PCR for detecting MLSB resistance genes, 5 (83.3%) were conducted in Egypt and 1 (16.7%) was in Uganda. The detection rate of inducible clindamycin resistance in MRSA and MSSA was not reported in Ethiopian studies and a study from Uganda. Additionally, the prevalence of the iMLSB phenotype was relatively higher in children and burn patients (Table 1).

## 4. The Geographic Area of Studies Reported Inducible Clindamycin-Resistant *S. aureus* in Africa

**Figure 2:**
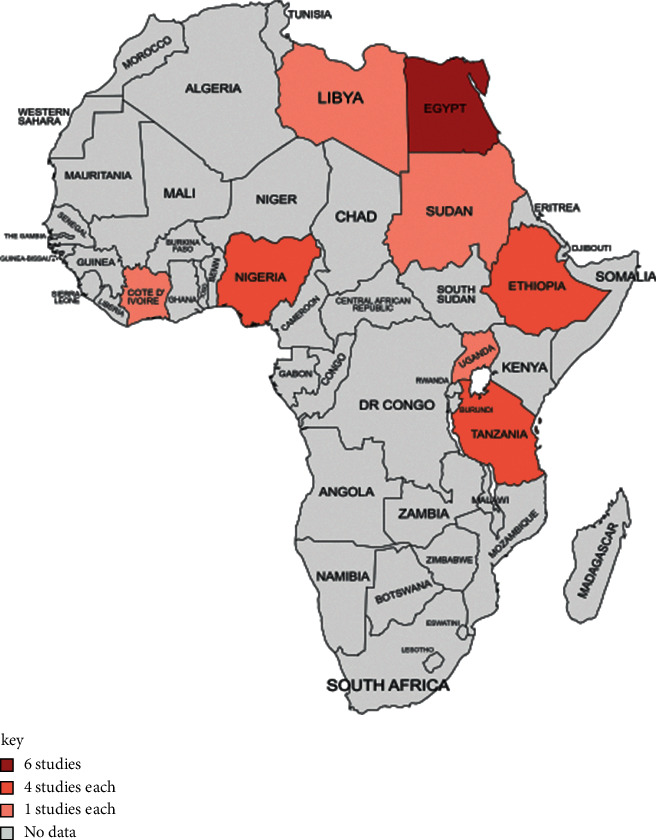
Map of Africa showing number of articles from eight countries (Libya, Egypt, Tanzania, Ethiopia, Nigeria, Sudan, Uganda, and Côte d'Ivoire) which reported inducible clindamycin-resistant *S aureus* (drawn from https://mapchart.net/africa.html).

The incidence of inducible clindamycin resistance in *S. aureus* isolates was reported in eight countries (Libya, Egypt, Tanzania, Ethiopia, Nigeria, Sudan, Uganda, and Côte d'Ivoire) in three geographic regions of Africa such as eastern (9 studies), northern (8 studies), and western (5 studies) regions based on United Nations classification (Figure 2). Most of the studies reporting inducible clindamycin resistance in *S. aureus* were conducted in Egypt (75%, 6/8), followed by Tanzania (50%, 4/8), Ethiopia (50%, 4/8), Nigeria (50%, 4/8), Libya, Sudan, Uganda, and Côte d'Ivoire (12.5%, 1/8) each (Figure 2).

### 4.1. The Prevalence of Inducible Clindamycin Resistance among *S. aureus* Isolates

In our review, we assessed the overall prevalence of inducible clindamycin resistance among *S. aureus* isolates by adding all iMLSB phenotypes and dividing it by the total number of *S. aureus* isolates. The total number of *S. aureus* isolates in the review was found to be 3064. Among the total *S. aureus* isolates, 605 had an iMLSB phenotype. Thus, the overall prevalence of inducible clindamycin resistance in this review was found to be 19.8%. Similarly, the overall prevalence of the iMLSB phenotype in MRSA was calculated by adding all the number of iMLSB phenotypes and dividing it by the total number of MRSA isolates, which was 26.8% (279/1041). Studies were conducted between 2007 and 2021 from different areas of the country [12–33].

**Figure 3:**
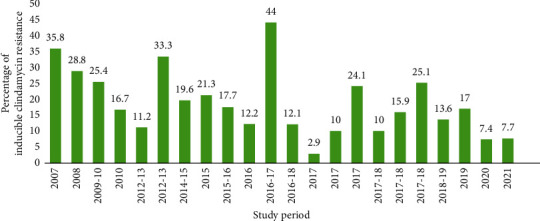
The trend of inducible clindamycin-resistant *S. aureus* in Africa from 2007 to 2021.

The prevalence of inducible clindamycin resistance among *S. aureus* isolates varies from place to place due to the difference in local clindamycin resistance. Inducible clindamycin resistance was first reported in 2007 in Libya among burn patients [12]. The prevalence range of inducible clindamycin resistance among the *S. aureus* isolates was 2.9–44% [12–33] (Figure 3). The highest peak prevalence of inducible clindamycin resistance among *S. aureus* isolates documented on the continent was 44.0% in 2017 in Egypt [21] and the minimum prevalence was 2.9% from Côte d'Ivoire [24]. In 2007 and 2013, respectively, the second (35.8%) [12] and third (33.3%) [17] highest peaks of inducible clindamycin resistance prevalence were reported. Despite a 33.3% record of inducible clindamycin resistance in 2013, there has been a progressive drop in the prevalence of inducible clindamycin resistance since 2007 [17]. A similar 10.0% prevalence was observed in Egypt during 2017 and 2018 [23, 27]. Generally, there were heterogeneous distribution and prevalence rate of inducible clindamycin resistance among *S. aureus* isolates in Africa according to the reviewed studies (Figure 3).

**Figure 4:**
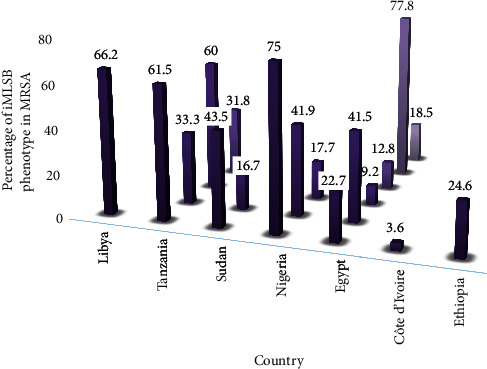
The prevalence of iMLSB phenotype in MRSA strains in African countries (ordered using study period: Libya (2007), Tanzania (2008 to 2015), Sudan (2009 to 2018), Nigeria (2012 to 2018), Egypt (2016 to 2019), Côte d'Ivoire (2017), and Ethiopia (2017)). Abbreviations: iMLSB, inducible macrolide-lincosamide-streptogramin B; MRSA, methicillin-resistant *Staphylococcus aureus*.

A high number of iMLSB phenotypes were observed in MRSA isolates, ranging from 3.6 to 77.8% than MSSA, which was within a range of 0–58.8% [12–16, 18–30] (Table 1). The highest prevalence of iMLSB phenotype among MRSA strains was reported in Egypt, 77.8% [29], followed by Nigeria, 75.0% [16]; Libya, 66.2% [12]; and Tanzania (61.5% [13]; 60.0% [18]). The lowest prevalence of the iMLSB phenotypes in MRSA strains was demonstrated in Côte d'Ivoire, 3.9% [24] (Figure 4). A zero prevalence of the iMLSB phenotype among MSSA strains was observed in 2007 and 2017 in Libya [12] and Côte d'Ivoire [24], respectively (Table 1). Additionally, cMLSB phenotype was reported in MRSA and MSSA strains, ranging between 0–75.0% and 0–60.0%, respectively [12, 13, 15, 19–30] (S1).

### 4.2. Resistance Genes Related to MLSB Resistance

Despite the lack of complete information on the resistance genes, investigations have shown that many *S. aureus* isolates coproduced resistance genes such as erm (A, B, C, E) genes, msrA genes, mphC genes, and lnuA genes. Among the recovered erm genes in Egypt, the ermC gene was a highly detected resistance gene, at 70.0% [23], followed by the ermA gene, with a 67.9% detection rate [21]. The msrA gene was also detected among *S. aureus* isolates, with a 70.0% high detection rate in Egypt [23]. A study in Uganda also revealed ermC genes (32.7%) [17]. Additionally, the mphC genes (40.0%), and lnuA genes (20.0%) were also detected from a study in Egypt [25] (Table 1).

## 5. Discussion

The phenotypic analysis of the inducible resistance in *S. aureus* to clindamycin was demonstrated through the *D*-test across African countries. In this review study, the prevalence of inducible clindamycin resistance among the *S. aureus* isolates was found to be 19.8%, ranging from 2.9 to 44.0% [12–33]. Various studies have reported a comparable proportion of inducible clindamycin resistance among *S. aureus* isolates obtained from human sources such as studies in Odisha state, eastern India, 22.0% [7]; Nepal, 23.4% [34]; Malaysia, 22.1% [35]; and Israel, 20.0% to 25.0% [36]. This finding is significantly higher than the findings of the iMLSB phenotype from the systematic review and meta-analysis in Iran with 10.4% overall prevalence [37]. In Indian studies, inducible clindamycin resistance rate was 5.2% in Kashmir valley [38], 7% in Assam [39], 15.2% in Chennai [40], 13.71% in the sub-Himalayan region [41], and 14.8% in central India [42]. In Nepal, lower inducible clindamycin resistance rate was indicated across different areas with a prevalence of 14.9% [43], 11.48% [8], 15.2% [44], and 12.1% [45]. While studies have also reported a high proportion of inducible clindamycin resistance as the prevalence in West Bengal, India, 41.3% [46]; Nepal (39.7% [47]; 34.8% [48]); Jordan, 76.7% [49]; and Tokyo, Japan, 91.0% [50]. The different iMLSB phenotypes observed in various studies are because of the variation in the study population, geographic region, the source of the specimen, methicillin susceptibility, usage of MLSB antibiotics in the community and hospital settings, and drug-resistant clones.

The emergence of inducible clindamycin resistance was comparatively higher among the clinical MRSA isolates as up to 77.8% recovered from cancer patients with febrile neutropenia [29]. In this study, the overall prevalence of inducible clindamycin resistance in MRSA strains was 26.8%. Comparable findings were reported in Nepal, 24.5% [44], and India (28.0% [41]; 25.0% [42]). Studies in Nepal (34.3% [47]; 76.4% [34]); India, 37.5% [51]; Malaysia, 46.7% [35]; and Jordan, 76.7% [49] demonstrated higher prevalence. However, this finding is higher than a study conducted in India (7.5% [39]; 18.7% [52]). The cMLSB phenotype prevalence in MRSA strain (28.9%) was in agreement with the study conducted in India, 29.26% [41], higher than studies conducted in Nepal (5.7% [47]; 11.2% [44]), India (16.6% [51]; 16.9% [39]), and Malaysia, 11.1% [35], but lower than a study from India, 64.8% [42]. This shows that clindamycin treatment proved effective against MSSA infections, but it can lead to treatment failure in MRSA infections, and iMLSB phenotypes can be mutated into cMLSB phenotype.

The most common mechanism for MLSB resistance in *S. aureus* is the target site modification of 23S ribosomal RNA mediated by erm genes and strains exhibiting the iMLSB phenotype having a high frequency of spontaneous constitutive resistance mutations. Regarding the genotypic confirmation of MLSB resistance genes, our findings demonstrated that erm (A, B, C, E) genes and msrA genes were commonly detected genes. This finding is supported by other studies [34, 49, 53–58]. This indicates that the high spread and transmission of these genes significantly contribute to the increasing acquiring clindamycin resistance in *S. aureus* strains. As a limitation, the inclusion of studies with lower sample size results in a bias in the finding. Most studies studied only the prevalence of inducible clindamycin resistance; only a few studies reported resistance genes.

## 6. Conclusion

The current review study demonstrated a high prevalence of inducible clindamycin resistance *S. aureus* isolates with varying proportions throughout the country. A relatively higher number of iMLSB phenotypes was observed in MRSA than in MSSA isolates and a high figure was reported in Egypt, 77.8%, and Nigeria, 75.0%. Additionally, these strains are closely related to resistance genes such as the ermA, ermC, and msrA genes. Hence, there is an urgent need for ongoing studies to further assess iMLSB-positive *S. aureus* strains especially MRSA and in the revision of clindamycin prescription. Genotypic detection of resistance genes is mandatory to minimize treatment failure.

## Figures and Tables

**Table 1:** Characteristics of studies reporting inducible clindamycin resistance in *S. aureus* isolates.

References	Study period	Country	Population	Specimen source	*S. aureus*	Detection method	iMLSB (%)	iMLSB in MRSA (%)	iMLSB in MSSA (%)	Resistance genes (%)
[12]	2007	Libya	Burn patients	Swabs from wounds, urine, blood, and others	120	*D*-test	35.8	43/65 (66.2)	0	—
[13]	2008	Tanzania	Surgical ward patients	Pus, wound swabs, and aspirates	160	*D*-test	28.8	16/26 (61.5)	30/134 (22.0)	—
[14]	2009 to 2010	Sudan	Healthcare workers and adult community	Nasal swabs	114	*D*-test	25.4	10/23 (43.5)	19/91 (20.9)	—
[15]	2010	Tanzania	Children under the age of five	Nasal swabs	114	*D*-test	16.7	4/12 (33.3)	15/102 (14.7)	—
[16]	2012 to 2013	Nigeria	Inpatient or outpatient	Vaginal, cervical, wound, ear, and eye swabs, urine, blood, semen, and others	205	*D*-test	11.2	3/4 (75.0)	20/34 (58.8)	—
[17]	2012 to 2013	Uganda	Inpatient or outpatient	Blood, CSF, swabs of ear, vaginal, nasal, pus, throat, urethral, urine, and wound	300	*D*-test and PCR	33.3	NA	NA	ermB (7.7), ermC (32.7), and msrA (14.3) genes
[18]	2014 to 2015	Tanzania	Surgery patients and HCWs	Nasal and wound swabs	189	*D*-test	19.6	6/10 (60.0)	31/179 (17.3)	—
[19]	2015	Tanzania	Inpatients	Nasal swabs	89	*D*-test	21.3	7/22 (31.8)	12/67 (17.9)	—
[20]	2015 to 2016	Nigeria	Poultry workers	Swabs from palm	186	*D*-test	17.7	13/31 (41.9)	20/155 (12.9)	—
[21]	2016	Egypt	Children	Pus, wound and eye swabs, blood, urine, catheter, respiratory sample, ear discharge, and others	230	D-test and PCR	12.2	15/66 (22.7)	13/165 (7.9)	ermA (67.9), ermB (3.6), ermC (12.3), and both ermA and ermC (3.6) genes
[22]	2016 to 2017	Egypt	Children	Blood, wound swab, and bronchoalveolar lavage	107	*D*-test and PCR	44.0	27/65 (41.5)	20/42 (47.6)	ermA (29.0), ermC (18.7, ermB (4.6), both ermA and ermC (1.0), and ermA, ermB, and ermC (3.7)
[23]	2016 to 2018	Nigeria	Inpatients or outpatients	Wound and abscess, blood, urine, ear, nasal, vaginal, and urethral swabs	265	*D*-test	12.1	29/164 (17.7)	3/100 (3.0)	—
[24]	2017	Côte d'Ivoire	Inpatients or outpatients	Pus, blood, pleural fluid, sputum, wound, and urine	35	*D*-test	2.9	1/28 (3.6)	0	—
[25]	2017	Egypt	Inpatients or outpatients	Endotracheal aspirates, sputum, blood, urine, and wound swabs	210	*D*-test and PCR	10.0	18/195 (9.2)	3/15 (20.0)	ermB (20.0), erm C (70.0), msrA (70.0), mphC (40.0), and lnuA (20.0) genes
[26]	2017	Ethiopia	Inpatients or outpatients	Wound swabs	79	*D*-test	24.1	16/65 (24.6)	3/14 (21.4)	—
[27]	2017 to 2018	Egypt	Inpatients or outpatients	Urine, pus, wound, wound swab, blood, and aspirates	100	*D*-test	10.0	9/70 (12.8)	1/30 (3.3)	—
[28]	2017 to 2018	Sudan	Inpatients	Postoperative wound swabs	94	*D*-test	15.9	7/42 (16.7)	8/52 (15.4)	—
[29]	2017 to 2018	Egypt	Cancer patients with febrile neutropenia	Pus, throat swabs, blood, urine, and sputum	179	*D*-test and PCR	25.1	35/45 (77.8)	10/45 (22.2)	ermE (33.3) ermC (15.6), and both ermC and ermE (51.0) genes
[30]	2018 to 2019	Egypt	Inpatients or outpatients	Pus, blood, tracheal aspirates, urine, ascetic and synovial fluid	176	*D*-test and PCR	13.6	20/108 (18.5)	4/68 (5.9)	ermA (16.0), ermB (45.5), and ermC (50.0) genes
[31]	2019	Ethiopia	Cancer patients	Nasal swabs	59	*D*-test	17.0%	NA	NA	—
[32]	2020	Ethiopia	Prisoners	Nasal swabs	27	*D*-test	7.4%	NA	NA	—
[33]	2021	Ethiopia	Adults with CAP	Sputum	26	*D*-test	7.7%	NA	NA	—

iMLSB, inducible MLSB; MRSA, methicillin-resistant *Staphylococcus aureus*; MSSA, methicillin-sensitive *Staphylococcus aureus*, PCR, polymerase chain reaction; CAP, community-acquired pneumonia; HCWs, healthcare workers; CSF, cerebral spinal fluid; NA, not available.

## Data Availability

All extracted data are freely available in the article and Supplementary Materials.

## Abbreviations

CLSI: Clinical and Laboratory Standards Institute
MLSB: Macrolide-lincosamide-streptogramin B
cMLSB: Constitutive MLSB
iMLSB: Inducible MLSB
MRSA: Methicillin-resistant *Staphylococcus aureus*
MSSA: Methicillin-sensitive *Staphylococcus aureus*
PCR: Polymerase chain reaction.

## Supplementary Materials

Supplementary Materials
*S1 Result*. The table shows the prevalence of cMLSB phenotype in MRSA and MSSA strains in African countries.
