# Supplementary material for: Inducible Clindamycin-Resistant Staphylococcus aureus Strains in Africa: A Systematic Review
Source: Int J Microbiol. 2022 Apr 19;2022:1835603. doi: 10.1155/2022/1835603 (PMC9042618; doi:10.1155/2022/1835603)
Supplement: Supplementary Materials — S1 Result. The table shows the prevalence of cMLSB phenotype in MRSA and MSSA strains in African countries. [file 1835603.f1.docx]

**S1 Result**

| **References** | **Country** | **cMLSB in MRSA (%)** | **cMLSB in MSSA (%)** |
| --- | --- | --- | --- |
| 12 | Libya | 6.2 | 0 |
| 13 | Tanzania | 3.7 | 0 |
| 15 | Tanzania | 0 | 2.0 |
| 19 | Tanzania | 9.1 | 1.5 |
| 20 | Nigeria | 16.7 | 9.1 |
| 21 | Egypt | 30.3 | 4.2 |
| 22 | Egypt | 50.8 | 40.0 |
| 23 | Nigeria | 27.4 | 27.7 |
| 24 | Côte d’Ivoire | 7.1 | NA |
| 25 | Egypt | 55.4 | 60.0 |
| 26 | Ethiopia | 3.08 | 0 |
| 27 | Egypt | 48.6 | 20.0 |
| 28 | Sudan | 5.0 | 11.5 |
| 29 | Egypt | 75.0 | 25.0 |
| 30 | Egypt | 51.9 | 17.6 |

**Table shows the prevalence of cMLSB phenotype in MRSA and MSSA strains in African countries (Libya, Tanzania, Sudan, Nigeria, Egypt, Côte d’Ivoire, and Ethiopia)**

cMLSB, constitutive macrolide-lincosamide-streptogramin B; MRSA, methicillin-resistant *Staphylococcus aureus*; MSSA, methicillin-sensitive *Staphylococcus aureus*
